# Supplementary material for: Prior exposure to long-day photoperiods alters immune responses and increases susceptibility to parasitic infection in stickleback
Source: Proc Biol Sci. 2020 Jul 1;287(1930):20201017. doi: 10.1098/rspb.2020.1017 (PMC7423467; doi:10.1098/rspb.2020.1017)
Supplement: WHITING_tables_figures_methods_ESM [file rspb20201017supp1.pdf]

Prior exposure to long day photoperiods alters immune responses and increases susceptibility to parasitic infection in stickleback

James R. Whiting*[1,2], Muayad A. Mahmud[1,3], Janette E. Bradley[1] and Andrew D.C. MacColl[1]

[1] School of Life Sciences, University of Nottingham, University Park, Nottingham, NG7 2RD, United Kingdom

[2] Department of Biosciences, University of Exeter, Geoffrey Pope Building, Exeter, EX4 4QD

[3] Scientific Research Center, Erbil Polytechnic University, Erbil, Iraq

Proceedings of the Royal Society B

DOI: 10.1098/rspb.2020.1017

Electronic Supplementary Material

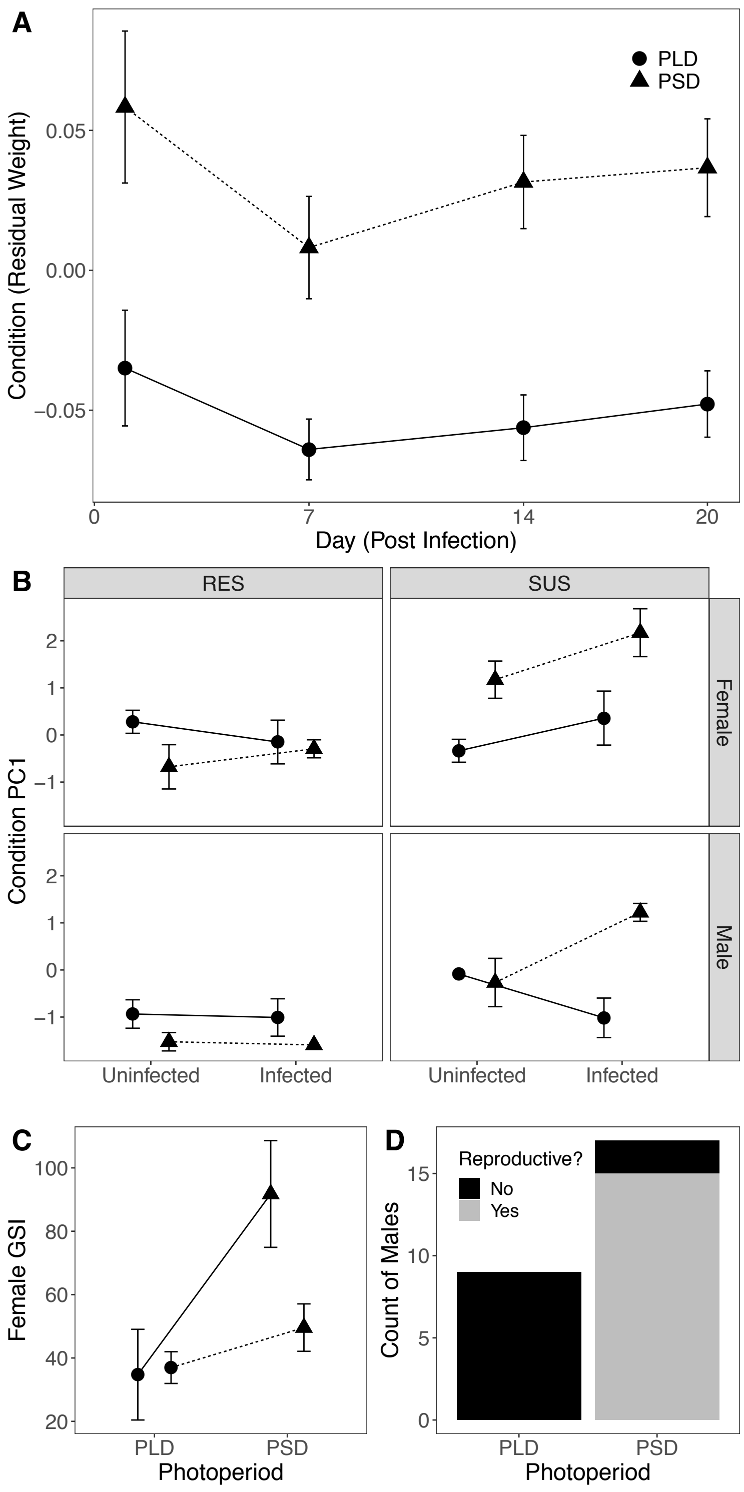

Figure S1: Experimental outcomes for measures of fish condition (**A**, **B**) and reproductive state for females (**C**) and males (**D**). For plots A and B, points show grouped means ± SE. Declines in condition with treatment are shown as reductions in residual weight (A) and a combined PC of HSI and ASI (**B**). Female GSI (**C**) lines are grouped by population rather than photoperiod (dashed = RES, solid = SUS) to illustrate consistent declines in GSI in PLD fish. Stacked bars in plot (**D**) shows counts of reproductive (black) and non-reproductive (grey) males in each photoperiod treatment.

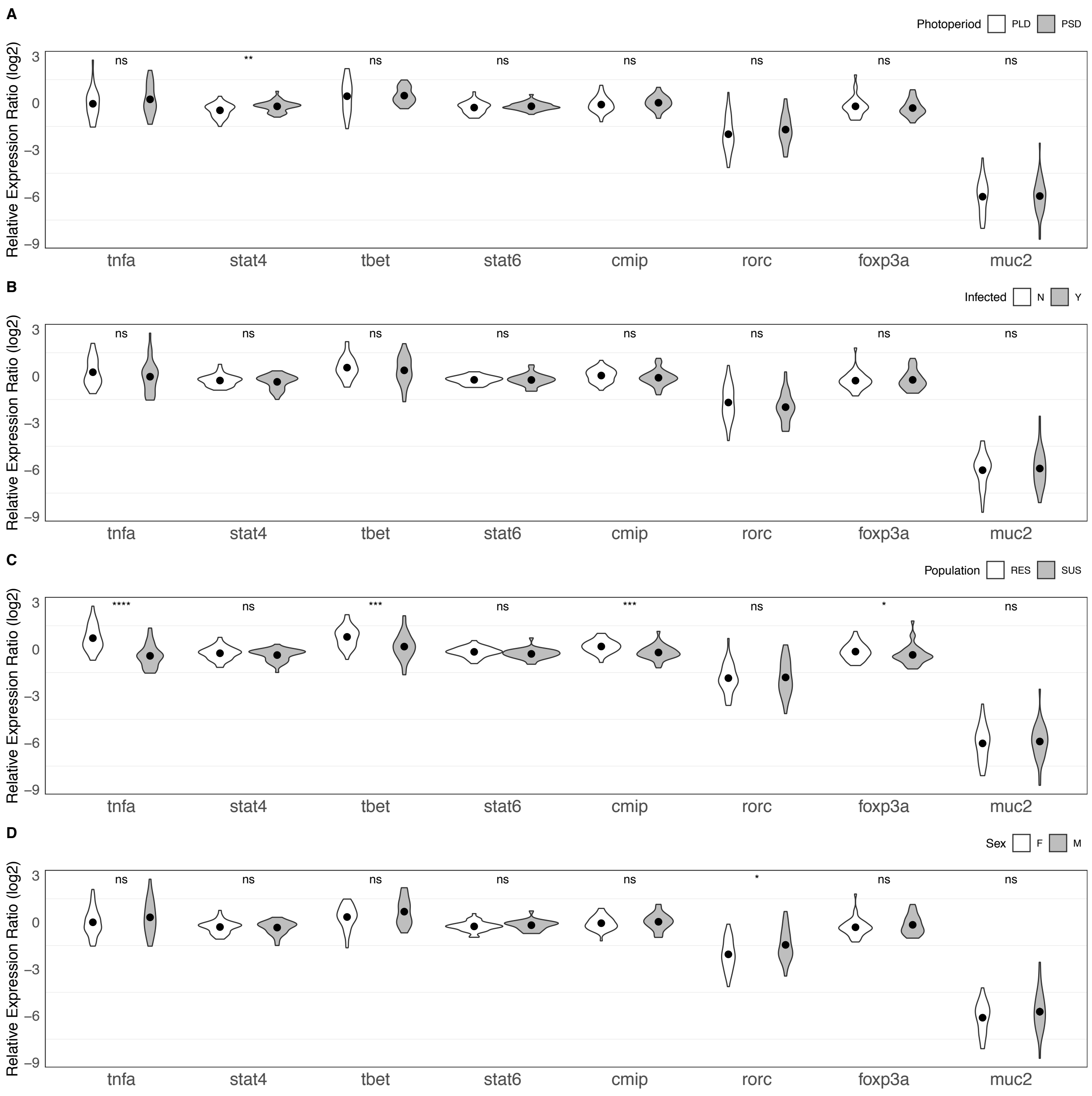

Figure S2: Log_2-transformed relative expression ratios for all genes in spleen tissues. Violins denote spread of variation with points for mean ± SE. Paired violins show variation between photoperiod treatments (**A**), infection treatments (**B**), source population (**C**), and sex (**D**). Annotations between paired violins represent results from tests of means (calculated through stat_compare_means in ggplot2) (ns = $p > 0.05$; * = $p < 0.05$; ** = $p < 0.01$; *** = $p < 0.001$).

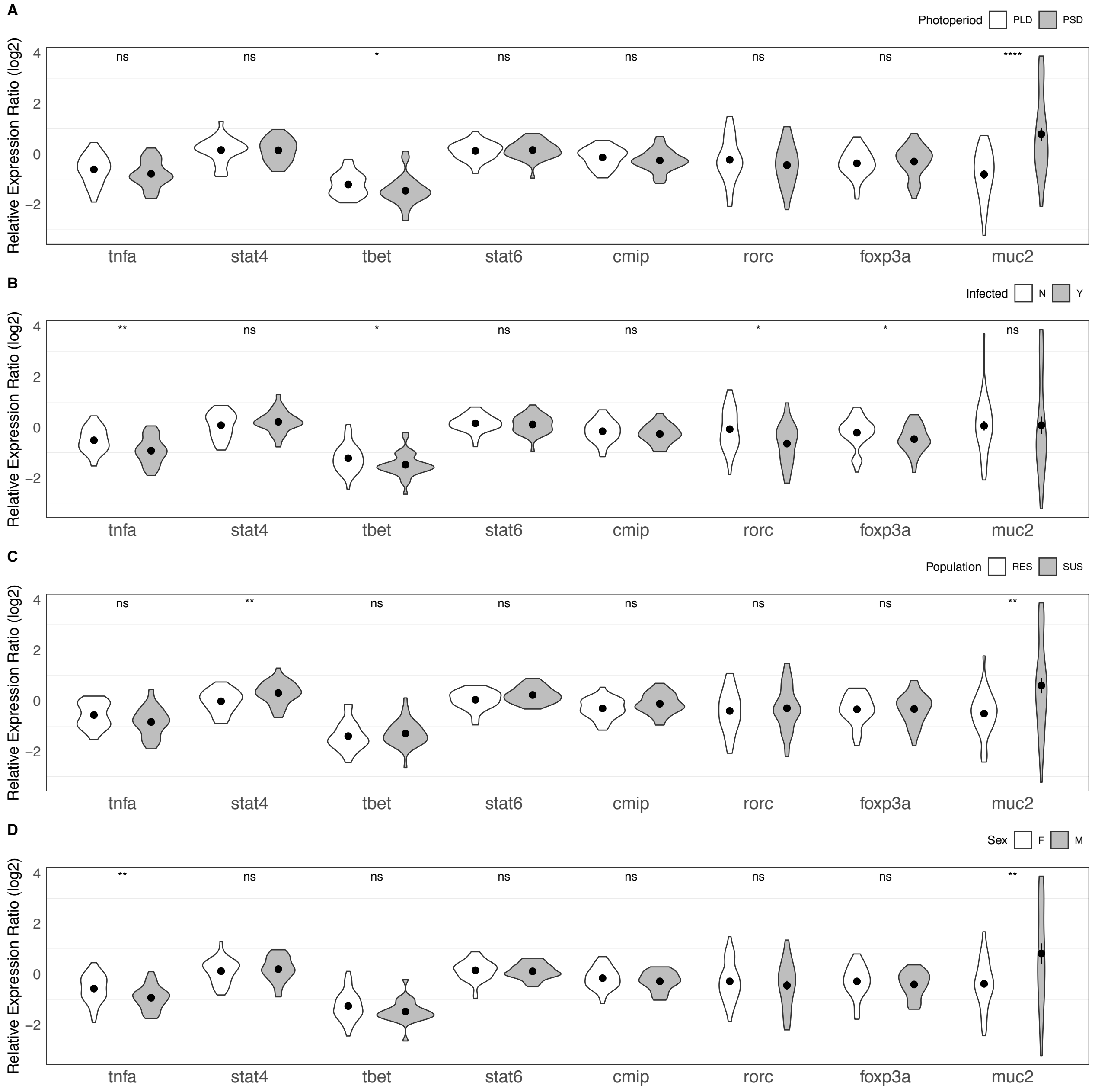

Figure S3: Log_2-transformed relative expression ratios for all genes in skin tissues. Violins denote spread of variation with points for mean ± SE. Paired violins show variation between photoperiod treatments (**A**), infection treatments (**B**), source population (**C**), and sex (**D**). Annotations between paired violins represent results from tests of means (calculated through stat_compare_means in ggplot2) (ns = $p > 0.05$; * = $p < 0.05$; ** = $p < 0.01$; *** = $p < 0.001$).

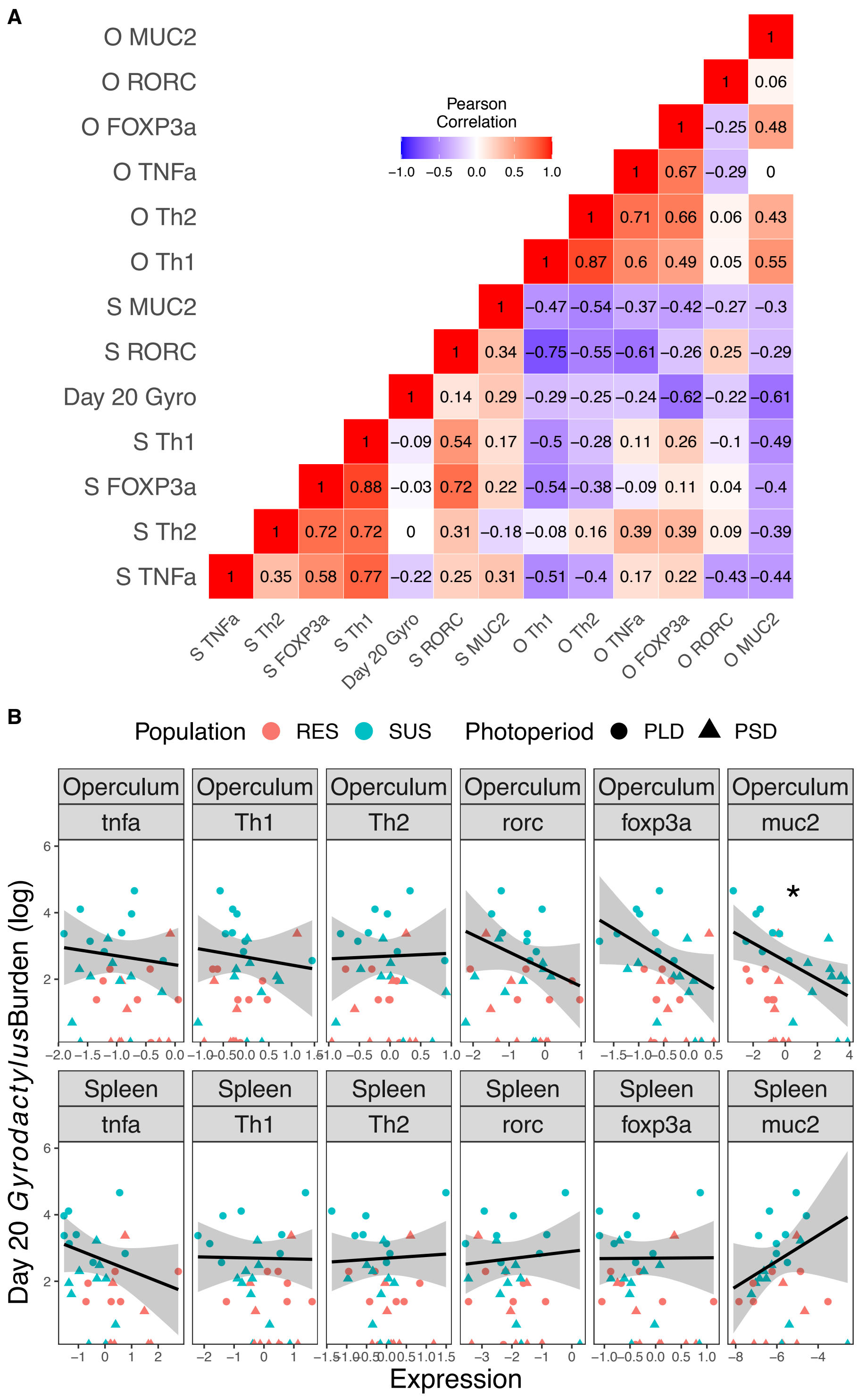

Figure S4: Correlation matrix for all immune variables (log_2-transformed relative expression ratios for genes or PC1 scores for Th1/Th2 variables) and day 20 *Gyrodactylus* burdens (**A**). Variables are ordered along axes to reveal highly correlated clusters. Colour of segments represents Pearson's correlation coefficient with exact coefficient given as a value. Logarithmic regressions between day 20 *Gyrodactylus* burdens and each of the 12 immune gene expression variables (**B**). Fitted negative binomial models are represented by lines with shaded 95% confidence intervals. Asterisks denote significance level of relationship (* < 0.05). Absence of asterisks denote non-significant linear relationships.

Table S1: Number of individuals across all possible treatment levels (16 cells).

	SUS Uninfected	RES Uninfected	SUS Infected	RES Infected
PLD Male	1	3	3	2
PLD Female	7	6	5	5
PSD Male	4	4	8	1
PSD Female	7	5	2	9

Table S2: Primer details for qPCR genes

Gene	ENSEMBL ID	Immune Role	Primer Sequence (5'-3')	Amplicon length
tnfα	ENSGACG00000013372	Pro-Inflammatory cytokine	Fwd-GCTTGGTTCTGGCCAGGTTT<br>Rev-GCTGCTGATTTGCCTCAACG	125
stat4	ENSGACG00000002684	Transcription factor for development of Th1 cells	Fwd-CTCTCAGTTTCGAGGCTTGCTT<br>Rev-GGCAGTTGGCTCACATTGG	100
tbet	ENSGACG00000003829	Transcription factor modulates expression of Th1-cell cytokines eg. IFNγ	Fwd-CACATCGTGGAGGTGAAGGA<br>Rev-CGGTGACGGCGATGAACT	99
cmip	ENSGACG00000002527	Signalling protein in Th2 pathway	Fwd-GGCATGGAGGTCGTCAAGAA<br>Rev-TAGCAGGAGTAAATGGCGGC	119
stat6	ENSGACG00000008477	Involved in mediating Th2 cytokines IL-4 and IL-3 signalling	Fwd-CTCAGCCACAGTTCCAACCGTTC<br>Rev-GTCGGATGTTCTGGACCTCGAGT	104
foxp3a	ENSGACG00000012777	Promotes development and function of Treg cells	Fwd-GTTGACCCATGCAATTCCGA<br>Rev-CTGCTGTAGTTGTGGTCCTG	94
rorc	ENSGACG00000012239	Promotes differentiation of pro-inflammatory Th17 cells	Fwd-TTGACTTTGCCCACGGTATG<br>Rev-TGCTGAACTCTGCCTCTGT	121
muc2	ENSGACG00000014109	Principal organic constituent of mucus	Fwd-AGAATGGCGAGTCCTGGAA<br>Rev-AGATGGGTTGTTGTGGTGTG	105

Table S3: Model effects for condition and *G. gasterostei*. Models were reduced from a maximum model including photoperiod treatment, infection group, population and sex (if applicable).

**Analysis**	**Response Variable**	**Factor[1]**	**Family**	**ΔAIC[2]**	***F***	**d.f.**	***P*[3]**	**Effect[4]**
Condition	Condition	Photoperiod * Population	Gaussian	26.62	31.23	1,64	**<0.001**	*NA*
	(PC1 of HSI and ASI)	Sex		21.5	24.7	1,64	**<0.001**	F > M
		Infection Group * Population		3.23	4.82	1,64	**0.03**	*NA*
		Infection Group * Photoperiod		1.89	3.55	1,64	0.06	*NA*
	Female GSI	Photoperiod * Population	Gaussian	4.3	6.17	1,42	**0.017**	*NA*
	(log10-transformed)	---						
		Infection Group		1.7	0.27	1,41	0.607	
Gyrodactylus	Day 20 pi burden	Photoperiod	Negative	4.01	6.71	1,32	**0.014**	PLD > PSD
		Population	Binomial	5.34	8.57	1,32	**0.006**	SUS > RES

		Sex		-2	0.002	1,31	0.962	
	Clearing Infection	Photoperiod	Binomial	3.64	3.9	1,33	0.057	PSD > PLD

		Sex		0.84	2.51	1,32	0.123	
		Population		-1.89	0.12	1,31	0.737	

[1] Variables are ordered such that those removed first from the model are lowest down. Variables included in final models are those above "---".
[2] Values show changes in AIC based on that variable being removed from the model. Typically, increases in AIC of >2 result in poorer fit. "NA" represents variables included in an interaction for which changes in AIC could not be assessed outside of the interaction.
[3] Significance at $p < 0.05$ is denoted in bold.
[4] Effects are included where factors are significant and outside of interactions.

Table S4: Model effects for immune gene expression variables. Models were reduced down from a maximum model including photoperiod treatment, infection group, population and sex (if applicable).

Gene / PC	Spleen						Operculum					
	Factor[1]	ΔAIC[2]	*F*	d.f.	*P*[3]	Effect	Factor[1]	ΔAIC[2]	*F*	d.f.	*P*[3]	Effect
tnfα	Population	34.09	45.349	1,66	**< 0.001**	RES > SUS	Infection Group	8.556	10.911	1,66	**0.002**	Uninfected > Infected
	Sex	5.24	7.295	1,66	**0.009**	M > F	Sex	5.336	7.404	1,66	**0.008**	F > M
	---						---					
	Photoperiod	0.88	2.772	1,65	0.101		Population	1.707	3.587	1,65	0.063	
	Infection Group	0.61	2.465	1,64	0.121		Photoperiod	-0.855	1.071	1,64	0.305	
foxp3a	Photoperiod * Infection Group	3.9	5.806	1,65	**0.019**		Photoperiod * Population	2.43	4.181	1,63	**0.045**	
	---						Photoperiod * Infected	9.89	11.851	1,63	**0.001**	
	Population	0.19	2.057	1,64	0,156		---					
	Sex	0.24	2.085	1,63	0.154		Sex	-0.08	1.751	1,62	0.191	
rorc	Sex	4.05	6.142	1,66	**0.016**	M > F	Infection Group	5.68	8.984	1,67	**0.004**	Uninfected > Infected
	---						---					
	Infection Group	0.31	2.246	1,66	0.139		Photoperiod	-0.64	1.308	1,66	0.257	
	Photoperiod	-1.29	0.667	1,65	0.417		Population	-1.36	0.615	1,65	0.436	
	Population	-1.98	0.026	1,64	0.874		Sex	-1.77	0.211	1,64	0.647	
muc2	Infection Group * Sex	4.85	6.783	1,65	**0.011**		Photoperiod * Population	8.68	10.217	1,61	**0.002**	
	---						Photoperiod * Infection Group	10.71	17.897	1,61	**< 0.001**	

	Population	-1.94	0.059	1,65	0.809		Photoperiod * Sex	15.75	12.338	1,61	**< 0.001**	
	Photoperiod	-1.99	0.006	1,64	0.937							
Th1 PC1	Photoperiod * Sex	2.5	4.313	1,65	**0.042**		Population	3.55	5.618	1,67	**0.021**	SUS > RES
	Population	6.42	8.305	1,65	**0.005**	RES > SUS	---					
	---						Photoperiod	0.21	2.148	1,66	0.148	
	Infection Group	-0.6	1.252	1,64	0.267		Sex	-1.39	0.576	1,65	0.451	
							Infection Group	-1.62	0.358	1,64	0.552	
Th2 PC1	Population	6.3	8.558	1,67	**0.005**	RES > SUS	Population	3.9	5.986	1,67	**0.017**	SUS > RES
	---						---					
	Sex	0.2	2.141	1,66	0.148		Sex	0.08	2.019	1,66	0.16	
	Photoperiod	-0.43	1.497	1,65	0.226		Infection Group	-0.98	0.967	1,65	0.329	
	Infection Group	-1.39	0.569	1,64	0.454		Photoperiod	-0.38	0.223	1,64	0.634	

[1]: Variables are ordered such that those removed first from the model are lowest down. Variables included in final models are those above "---".
[2]: Values show changes in AIC based on that variable being removed from the model. Typically, increases in AIC of >2 result in poorer fit. "NA" representsvariables included in an interaction for which changes in AIC could not be assessed outside of the interaction.
[3]: Significance at $p < 0.05$ is denoted in bold.

Supplementary Methods

Extended detail of infection protocol

On the morning of infection, donor stickleback were collected from Tottle Brook on the University of Nottingham campus (52°55'58.4"-1°12'05.8"), which are infected with *Gyrodactlyus gasterostei*. The use of *G. gasterostei* rather than *G. arcuatus* removed any possible effects of host local adaptation or host-parasite coevolution.

Donor fish were euthanized by an overdose of MS222 (400 mg L^{-1}) in accordance with Home Office regulations. Gyrodactylids were collected in a single petri dish by allowing worms to detach from clipped fins left in dechlorinated water for 10 minutes. This minimised the likelihood of parasites coming from the same individual host. Study fish were anaesthetised by a low-dose of MS222 (280 mg L^{-1}) and had their weight and standard length recorded. Fish were infected in a random order by placing the caudal fin in close proximity with donated worms in a petri dish, under a low-powered microscope until they attached; each fish was infected with three worms. Fish from the uninfected treatment group were exposed to the same dosage of MS222 for 30 seconds and were weighed, measured and handled to simulate the infection protocol.

Full details of molecular methods – RNA extraction, reverse-transcription and qPCR

Sampling order was randomised, and RNA was extracted from whole spleens and opercula (stored in RNAlater) using the Genejet RNA purification kit (Thermo Scientific) according to the manufacturer's protocol.

RNA purity was assessed on a NanoDrop1000 spectrophotometer (Thermo Scientific). RNA integrity was assessed following DNase (Primerdesign) treatment, following the manufacturer's protocol, by visualisation of 4 µl of sample on a 2% agarose gel stained with ethidium bromide.

Reverse transcription was performed using nanoScript2 RT kit (Primerdesign) according to the manufacturer's protocol using approximately 1.5 µg of template. This protocol uses a combination of random nonamer and oligo-dT priming. Genomic DNA contamination was assessed via light PCR using intron-spanning primers. All cDNA samples were diluted 1:10 with nuclease free water before use.

A total reaction volume of 10 µl was used to perform qPCR reactions consisting of 5 µl of PrecisionFAST low ROX mastermix with SYBR green (Primerdesign), 2.5 µl of nuclease-free water, 0.25 µl of each primer at working concentration and 2 µl of cDNA template. Reactions were performed in 96-well optical PCR plates with optical seals (StarLab) in an ABI 7500 Fast real-time thermocycler (Applied Biosystems). Samples were incubated at 95 °C for 20 s, followed by 45 cycles of 95 °C for 3 s and 60 °C for 30 s. A melt-curve analysis was also included to confirm product.

A custom stickleback with SYBR geNorm analysis was conducted to select appropriate housekeeping genes for this experiment for each tissue type. This analysis chooses the most stably expressed pair of 6 housekeeping genes for normalisation of expression for genes of interest. The analysis was conducted per the manufacturer's protocol using 15 randomly selected samples, consisting of all combinations of sex, population, photoperiod and infection treatment, with the exception of an PSD infected SUS male. This combination was omitted randomly due to plate size constraints. Of the 6 candidate reference genes supplied (*b2m*, *gapdh*, *rpl13a*, *hprt1*, *tbp* and *top1*) *b2m* and *rpl13a* were the most stably expressed combination for spleens and *hprt* and *tbp* were the most stably expressed combination for opercula.

In total, 69 spleen and 69 operculum samples were analysed. A reference sample, comprised of a pool of all 138 samples, was made up and used as a control reference across all plates. 3 plates were run per gene. Well position for each duplicate was randomly assigned. In total, 8 genes were amplified using 6 primers published in Robertson et al.(1) and 2 primers (*rorc*, *muc2*) designed and tested by Primerdesign Ltd. Genes for which assays had been previously developed (1). Relative

expression values were calculated per the ΔΔCq method(2) and adjusted for the amplification efficiencies of each primer pair. Expression values were standardized against the tissue-specific geometric mean Cq of two reference genes.

Choice of assay genes

The pro-inflammatory gene *tnf*α is a key component of innate immunity in teleosts and other vertebrates (3,4), activating macrophages, eliciting inflammation and increased respiratory burst activity.

The Th1 transcription factor STAT4 (*stat4*) promotes the differentiation of Th1 cells and has been identified in teleost genomes. STAT4 activates the expression of *tbet*, which in mammals represents the master transcription factor for Th1 cell differentiation, activating other genes required to promote differentiation and suppressing the development of other T-cell lineages such as Th2 and Th17 (5). Fish *tbet* has been cloned in a number of teleosts and its existence and function are supported by conserved synteny across teleosts and humans and expression studies demonstrating its importance in fish T cell-mediated immunity (5). Expression of Th1-associated genes in infection studies suggest that fish may possess a full and conserved Th1 pathway (4). Th1 adaptive immunity is associated with intracellular parasites, whilst Th2 adaptive immunity is associated with extracellular infection. Th2 cell differentiation is associated with up-regulation of *cmip* and *stat6* in mammals, which is also upregulated alongside other markers of Th2 responses in zebrafish head kidney and spleen cells in response to immunostimulation (6).

Th17 cells, a subset of pro-inflammatory T cells, are regulated by the transcription factor ROR-γt (*rorc*) in mammals, which increases in mRNA levels during the differentiation of naïve CD4+ lymphocytes into Th17 cells (7). *rorc* is present in fish genomes, albeit in several isoforms (5) along

with the other major components of Th17 cell development, however expression studies in trout both support and contradict the notion that *rorc* in fish behaves like its mammalian counterpart (8)

Mucins represent constituent molecules of mucosal immunity and are secreted within mucus, forming a protective gel-like layer to protect epithelial surfaces (9). The mucin gene *muc2* is typically expressed at intestinal and respiratory surfaces and has been characterised in carp (10), showing high similarity to its mammalian and avian equivalent. Of specific interest here, mucin genes exhibit strong divergence between marine and freshwater stickleback, suggesting significant ecological associations with adaptation (11).

Treg activity, which regulates the immune response in general, is characterised by *foxp3a* expression. This gene has been identified in many teleost genomes and has demonstrable roles in immune regulation in fish and mammals (4). The genes included in this study therefore serve to capture variation across a range of immune responses; specifically, in the expression of an innate inflammatory cytokine (*tnf*α); markers for T-cell differentiation for Th1 adaptive (*stat4, tbet*), Th2 adaptive (*cmip, stat6*), Th17 adaptive (*rorc*) responses; mucin production as a measure of mucosal immunity (*muc2*); and Treg cytokine activity (*foxp3a*).

Data analysis

All data were analysed in R version 3.3.2 (12).

Effect of photoperiod/infection on fish condition

Condition was analysed in two ways. Condition over the infection period was tracked weekly by measuring residual weight. Residual weight was calculated as the deviation from weight predicted by standard length from the relationship between ln-transformed weight prior to the infection period and standard length (with additional model effects to control for POP and SEX variation in the

weight x length relationship). We therefore tracked residual weight of individuals across the infection period and assessed how this relationship was affected by PHOTO and INFECTED using linear mixed models (LMM).

We used the first principal component ("CONDITION") of HSI and ASI as an approximation of fish condition at day 20 pi as the two were correlated (Spearman's R = 0.458, $p < 0.001$)). CONDITION was modelled using GLMs as above using a Gaussian distribution and identity link and variables were selected from an initial model consisting of PHOTO, POP, INFECTED and SEX. Significance of remaining variables was inferred through ANOVA F-tests.

Female GSI was $\log_{10}$-transformed to meet the assumptions of a Gaussian fitted GLM, with the most complex model comprised of PHOTO, POP, and INFECTED. Males were divided into those that were fully reproductive (REPRO = 3) and those that were not (REPRO < 3). A binomial-fitted GLM was deemed inappropriate due to the extreme biasing effect of PHOTO, therefore a contingency table and chi-squared test was used to assess the reproductive status of males from each room.

Effect of photoperiod/infection on infection dynamics

Gyrodactylus burdens were tracked over the 20-day infection period weekly. The relationship between infection dynamics and time was assessed for interactions with PHOTO, POP and SEX using LMMs with a negative binomial family.

To assess the effect of treatment group on peak (20 days pi) *Gyrodactylus* burdens of infected fish, we used Generalized Linear Models (GLMs) fitted with a negative binomial distribution and logarithm link function. These were shown to fit the data better than models with Poisson distribution. We also used GLMs with binomial errors ('cleared' = 1, 'infected' = 0) to model the probability that individual fish cleared their infection. Models were fitted with the independent variables PHOTO, POP and SEX. Variables were modelled additively and removed sequentially using a

top-down approach. Biologically plausible interactions between independent variables were assessed at each level to see if model fit could be improved. If model fit was significantly improved interactions were added, and top-down model selection was continued until the simplest, best fitting model was found. Model fit was assessed through AIC (Akaike Information Criterion) and residual diagnostic plots. The significance of remaining factors was inferred through Wald's Tests using the R package 'survey'

Effect of photoperiod/infection on relative expression of immune genes

Relative expression values were log_2-transformed to account for the inherent skewness of relative expression data. Genes associated with Th1 and Th2 adaptive immunity were grouped through covariance-matrix based PCA, with PC1 retained for each. In total therefore, we analysed 12 immune variables: *tnfα*, *foxp3a*, *rorc*, *muc2*, Th1 PC1 and Th2 PC2 across spleen and opercula tissues.

We fitted Gaussian family GLMs to each expression variable to understand causes of variation in the way that genes were expressed in all fish. The maximum models included the treatment variables photoperiod group ("PHOTO") and infection group ("INFECTED") and the control variables of population ("POP") and sex ("SEX"). Models were fitted by the above methods and the significance of remaining factors was inferred through ANOVA F-tests, and post-hoc Tukey tests of significant interaction terms using the R package *lsmeans*.

The relationships between the 12 immune gene expression variables and *Gyrodactylus* infection were modelled individually using GLMs with negative binomial distributions. Day 20 burdens were modelled as the dependent variable in each case. Significance of relationships was determined through Wald's tests as above.

References

1. Robertson S, Bradley JE, MacColl ADC. Measuring the immune system of the three-spined stickleback - investigating natural variation by quantifying immune expression in the laboratory and the wild. Mol Ecol Resour. 2016;16(3):701–13.

2. Pfaffl MW. A new mathematical model for relative quantification in real-time RT-PCR. Nucleic Acids Res [Internet]. 2001;29(9):45e–45. Available from: https://academic.oup.com/nar/article-lookup/doi/10.1093/nar/29.9.e45

3. Uribe C, Folch H, Enriquez R, Moran G. Innate and adaptive immunity in teleost fish: a review. Vet Med (Praha). 2011;56(10):486–503.

4. Secombes CJ, Wang T. The innate and adaptive immune system of fish. In: Infectious Disease in Aquaculture. Elsevier; 2012. p. 3–68.

5. Wang T, Secombes CJ. The cytokine networks of adaptive immunity in fish. Fish Shellfish Immunol. 2013;35(6):1703–18.

6. Mitra S, Alnabulsi A, Secombes CJ, Bird S. Identification and characterization of the transcription factors involved in t-cell development, t-bet, stat6 and foxp3, within the zebrafish, danio rerio. FEBS J. 2010;277(1):128–47.

7. Yang XO, Pappu BP, Nurieva R, Akimzhanov A, Kang HS, Chung Y, et al. T Helper 17 Lineage Differentiation Is Programmed by Orphan Nuclear Receptors RORα and RORγ. Immunity. 2008;28(1):29–39.

8. Monte MM, Wang T, Costa MM, Harun NO, Secombes CJ. Cloning and expression analysis of two ROR-γ homologues (ROR-γa1 and ROR-γa2) in rainbow trout *Oncorhynchus mykiss*. Fish Shellfish Immunol. 2012;33(2):365–74.

9. Linden SK, Sutton P, Karlsson NG, Korolik V, McGuckin MA. Mucins in the mucosal barrier to infection. Mucosal Immunol [Internet]. 2008;1(3):183–97. Available from: http://www.nature.com/doifinder/10.1038/mi.2008.5

10. van der Marel M, Adamek M, Gonzalez SF, Frost P, Rombout JHWM, Wiegertjes GF, et al. Molecular cloning and expression of two β-defensin and two mucin genes in common carp (*Cyprinus carpio* L.) and their up-regulation after β-glucan feeding. Fish Shellfish Immunol. 2012;32(3):494–501.

11. Jones FC, Chan YF, Schmutz J, Grimwood J, Brady SD, Southwick AM, et al. A genome-wide SNP genotyping array reveals patterns of global and repeated species-pair divergence in sticklebacks. Curr Biol. 2012;22(1):83–90.

12. R Core Team. R: A Language and Environment for Statistical Computing [Internet]. Vol. 0, R Foundation for Statistical Computing Vienna Austria. 2016. p. {ISBN} 3-900051-07-0. Available from: http://www.r-project.org/
